# Molecular survey of piroplasm species from selected areas of China and Pakistan

**DOI:** 10.1186/s13071-018-3035-x

**Published:** 2018-08-07

**Authors:** Muhammad Adeel Hassan, Junlong Liu, Muhammad Rashid, Naveed Iqbal, Guiquan Guan, Hong Yin, Jianxun Luo

**Affiliations:** 1 State Key Laboratory of Veterinary Etiological Biology, Key Laboratory of Veterinary Parasitology of Gansu Province, Lanzhou Veterinary Research Institute, Chinese Academy of Agricultural Sciences, Xujiaping 1, Lanzhou, Gansu 730046 People’s Republic of China; 2 Jiangsu Co-Innovation Center for the Prevention and Control of Important Animal Infectious Disease and Zoonose, Yangzhou University, Yangzhou, 225009 People’s Republic of China

**Keywords:** *Theileria*, *Babesia*, PCR, China, Pakistan

## Abstract

**Background:**

Piroplasmosis is an important animal disease that is a major constraint to the development of the livestock industry, often resulting in significant economic losses. Therefore, there is an urgent need to further understand the etiology of this and other tick-borne infections.

**Methods:**

Blood samples were collected from tick-infested animals from the Chakwal, Jhang, and Faisalabad districts of Punjab, Pakistan and from peri-urban areas around Hohhot, Inner Mongolia, China to investigate the presence of *Babesia* and *Theileria* species. In total, 450 blood samples were collected with FTA cards from cattle of the study areas of Pakistan; the genomic (g)DNA of one hundred and twenty samples from cattle in Inner Mongolia were provided by the Lanzhou Veterinary Research Institute, China. Following the extraction of gDNA, the *18S* rRNA gene (V4 hypervariable region) of piroplasms was amplified in all samples using semi-nested PCR. Positively identified samples were sequenced for the identification of *Theileria* and *Babesia* species. The partial full-length sequence of *18S* rDNA was amplified for species confirmation of *Theileria*-positive samples, whereas the *RAP-1c* gene was amplified for *Babesia bigemina*-positive samples.

**Results:**

Semi-nested PCR results revealed that 144 (25.26%) samples were positive for piroplasms. *Theileria annulata* was the most prevalent species (115/144; 20.17%), followed by *Theileria orientalis* (16/144; 2.80%). Among *Babesia*, the only species recorded was *Babesia bigemina* (13/144; 2.28%).

**Conclusion:**

The present study reveals new data on the prevalence of piroplasm species in bovine populations of selected areas of China and Pakistan and their phylogenetic relationships. It is also the first detailed report of *T. orientalis* from native animals in Pakistan.

## Background

The livestock industry is an important part of the economy in Pakistan, contributing 11.6% of the total gross domestic product during the economic year 2016–2017. According to statistics, there are 82.1 million heads of cattle and buffalo in Pakistan [1]. Although it is estimated that two thirds of the human population of Pakistan is involved in the livestock sector either directly or indirectly, management practices and disease prevention and control strategies are poorly developed.

Piroplasmosis is one of the most important diseases, constraining the livestock industry in tropical and subtropical areas of the world [2]. It is caused by species of two genera of haemoprotozoa, *Theileria* and *Babesia*. In general, *Theileria annulata* and *Theileria parva* are considered to be the most pathogenic species, causing theileriosis in cattle and buffalo populations of tropical and subtropical areas, whereas *Theileria mutans* and *Theileria orientalis* are responsible for asymptomatic disease [3]. Hard ticks of the genera *Hyalomma*, *Rhipicephalus*, *Haemaphysalis* and *Amblyomma* are vectors of these *Theileria* spp., resulting in fatal economic losses in endemic areas of Asia and Africa [4, 5]. Clinical manifestations of theileriosis include: increased body temperature (40–41.5 °C), followed by depression, watery secretion from the eyes, nasal discharge, inflammation of the lymph nodes and anemia [6].

Bovine babesiosis is mainly caused by haemoprotozoans of the genus *Babesia*, including *Babesia bovis*, *Babesia divergens*, *Babesia ovata*, *Babesia major* and *Babesia bigemina*. Among these, *B. bovis* and *B. bigemina* are the most pathogenic species, transmitted by *Rhipicephalus microplus*, which is a globally distributed tick species. The other species, *B. ovata* and *B. major*, are transmitted by *Haemaphysalis longicornis* and *Haemaphysalis punctata*, respectively [7]. The clinical signs of babesiosis include fever, icterus, hemoglobinuria and anemia [8].

Diagnosis of the piroplasm species is usually done using Giemsa’s-stained blood smears, but this method cannot be utilized to identify carriers of the parasites, because the levels of parasitemia are low [9]. Studies show that carrier animals can be source of infection for a long time [10]. Serological assays have also been developed for detecting these parasite infections. Compared with other serological assays, such as indirect fluorescent antibody tests and compliment fixation tests, enzyme-linked immunosorbent assays are highly sensitive and easy to standardize, and, thus, are a preferred method for epidemiological studies [11, 12]. However, low specificity and false negative results in the case of carrier animals can hinder the efficacy of these tests [13]. However, for epidemiological surveys, it is recommended to detect carrier animals [14]. Molecular techniques can used comparatively reliably to determine the species spectrum of parasites in population studies, and species-specific PCR assays have been established for this purpose. Meanwhile, the development of reverse line blot (RLB) hybridization has enabled the simultaneous detection of various piroplasm species [15, 16]. PCR has been proven to be more specific and sensitive method for the identification of carrier and diseased animals in various studies worldwide.

In Pakistan, most published studies report the use of Giemsa’s staining for the detection of piroplasm species [5]. However, there have also been a few reports utilizing molecular techniques for diagnosis. For example, *T. annulata* has been detected by using species-specific primers in PCR [8, 17–19], but no confirmation of the results by DNA sequencing was provided. Gebrekidan et al. [20] reported the presence of *T. orientalis* in Pakistan for the first time from imported and native animals, reporting no *T. orientalis* in native animals by conventional PCR and low prevalence (6%) in native cattle as compared with imported animals (25.4%) using multiplexed tandem PCR. Recently, Hassan et al. [21, 22] determined the presence of *Theileria* spp. by using recombinase polymerase amplification. In terms of the detection of *Babesia* spp., PCR has been used in epidemiological studies reporting the presence of *B. bovis* and *B. bigemina* [8, 23, 24]. In the present study, semi-nested PCR was utilized to identify several species of piroplasm at the molecular level.

## Methods

### Sample collection

During July and August 2016, 450 blood samples were randomly collected from tick-infested but asymptomatic cattle in the Chakwal, Jhang and Faisalabad districts in Punjab, Pakistan. Blood was collected from the jugular vein using 10-ml disposable syringes and transferred to the four circles of a Whatman® FTA card (GE Healthcare Limited, Buckinghamshire HP7 9NA, UK) and allowed to air-dry. The FTA cards were shipped to Lanzhou Veterinary Research Institute, Chinese Academy of Agricultural Sciences Lanzhou, China for further processing. In addition, 120 samples of genomic (g)DNA collected from the blood of cattle in the field from Inner Mongolia, China were provided by the Vectors and Vector Borne Diseases Laboratory, Lanzhou Veterinary Research Institute, China.

### DNA extraction

Genomic DNA was extracted from the FTA cards using the genomic DNA purification kit (Qiagen, Hilden, Germany) according to the manufacturer’s instructions. The DNA concentration was determined with a NanoDrop 2000 spectrophotometer (Nanodrop Technologies®, Wilmington, DE, USA). DNA was stored at -20 °C until further analysis.

### PCR amplification

Semi-nested PCR was used to amplify the V4 hypervariable region of the *18S* rRNA gene from the gDNA of cattle. For this purpose, universal primers were used that can amplify several *Babesia* and *Theileria* spp. [25]. The first PCR reaction was conducted using primers RLB-F2 and RLB-R2, as shown in Table 1. The reactions were performed in a final volume of 25 μl containing 12.5 μl Premix Taq DNA polymerase (TaKaRa, Beijing, China), 1.0 μM of each primer, and 2 μl of DNA template. The second PCR was carried out with the primer RLB-FINT (as shown in Table 2) used as the forward primer, together with RLB-R2. The reaction mixture was as in the first PCR reaction, except the template was replaced by 2 μl of the first PCR product. PCR products were electrophoresed on a 1.5 % agarose gel and visualized under UV light. Positive PCR products were excised from the gel and purified using a BIOMIGA EZgene^TM^ Gel/PCR Extraction Kit (San Diego, CA USA). The DNA fragment was cloned into pGEM-T Easy Vector (Promega, Madison, USA). *Escherichia coli* Trans 5α (TaKaRa, China) was transformed and plasmid DNA from the selected clones was identified using PCR with the set of primers T7 (5'-TAA TAC GAC TCA CTA TAG GG-3') and SP6 (5'-ATT TAG GTG ACA CTA TAG-3') to verify the presence of correct inserts in selected clones before proceeding with the sequencing process. The reaction cycling in the second PCR was optimized with primer annealing at 54 °C for 30 s. The correct inserted clones were shipped to Genescript® (Shanghai, China) for sequencing [26].

**Table 1:** Details of the primers used in this study

Primer Name	Sequence (5'-3')	Product size (bp)	Annealing T (°C)	Reference
RLB-F2	GACACAGGGAGGTAGTGACAAG	393	52	[25]
RLB-R2	CTAAGAATTTCACCTCTGACAGT			
RLB-Fint	GACAAGAAATAACAATACRGGGC			
NBab-1F	AAGCCATGCATGTCTAAGTAGAAGCTTTT	~1600	58	[26]
18SRev-BT	GAATAATTCACCGGATCACTCG			
B.birap-1cseq-F	TTACGCTGCTTACTACAGCTTCA	1054	57	[7]
B.birap-1cRc	TTACGACGATCGTTTGAAGTACTTC			

*Abbreviation*: *T* temperature

**Table 2:** Detection of piroplasm species from selected areas of China and Pakistan using semi-nested PCR

Area	*T. annulata*	*T. orientalis*	*B. bigemina*	Negative	Total
	*n* (%)	*n* (%)	*n* (%)	*n* (%)	*n*
Chakwal	88 (46.80)	12 (6.38)	10 (5.31)	78 (41.48)	188
Jhang	5 (2.96)	0 (0)	0 (0)	164 (97.04)	169
Faisalabad	10 (10.75)	2 (2.15)	1 (1.07)	80 (86.02)	93
Inner Mongolia	12 (10.00)	2 (1.67)	2 (1.67)	104 (86.67)	120
Total	115 (20.17)	16 (2.80)	13 (2.28)	426 (74.73)	570

To confirm the *Theileria* spp., a long fragment of the *18S* rRNA gene (~1600 bp) was amplified from samples that were positive in short sequence amplification. The long fragment of the *RAP-1c* gene of *B. bigemina* (1054 bp) was amplified for species confirmation. The primers used are listed in Table 1.

### Phylogenetic analysis

The obtained sequences were aligned using the MegAlign component of the DNAStar software program (Version 4.0 DNAStar, Madison, USA). After alignment with related *Theileria* and *Babesia* spp. *18S* rDNA sequences retrieved from GenBank, parts of the cloning vector region were removed manually. The resulting sequences were then submitted to the GenBank database. A phylogenetic tree was generated based on the cloned sequences and the related *Theileria/Babesia 18S* rDNA sequences in GenBank by using the neighbor-joining algorithm in the MEGA 6.0 software [27]. The evolutionary distances were computed using the Kimura two-parameter method [28]. The phylogenetic trees based on long fragment of *18S* rRNA gene of *Theileria* spp. and *RAP-1c* gene of *B. bigemina* were generated using the above-mentioned procedure.

## Results

**Fig. 1:**
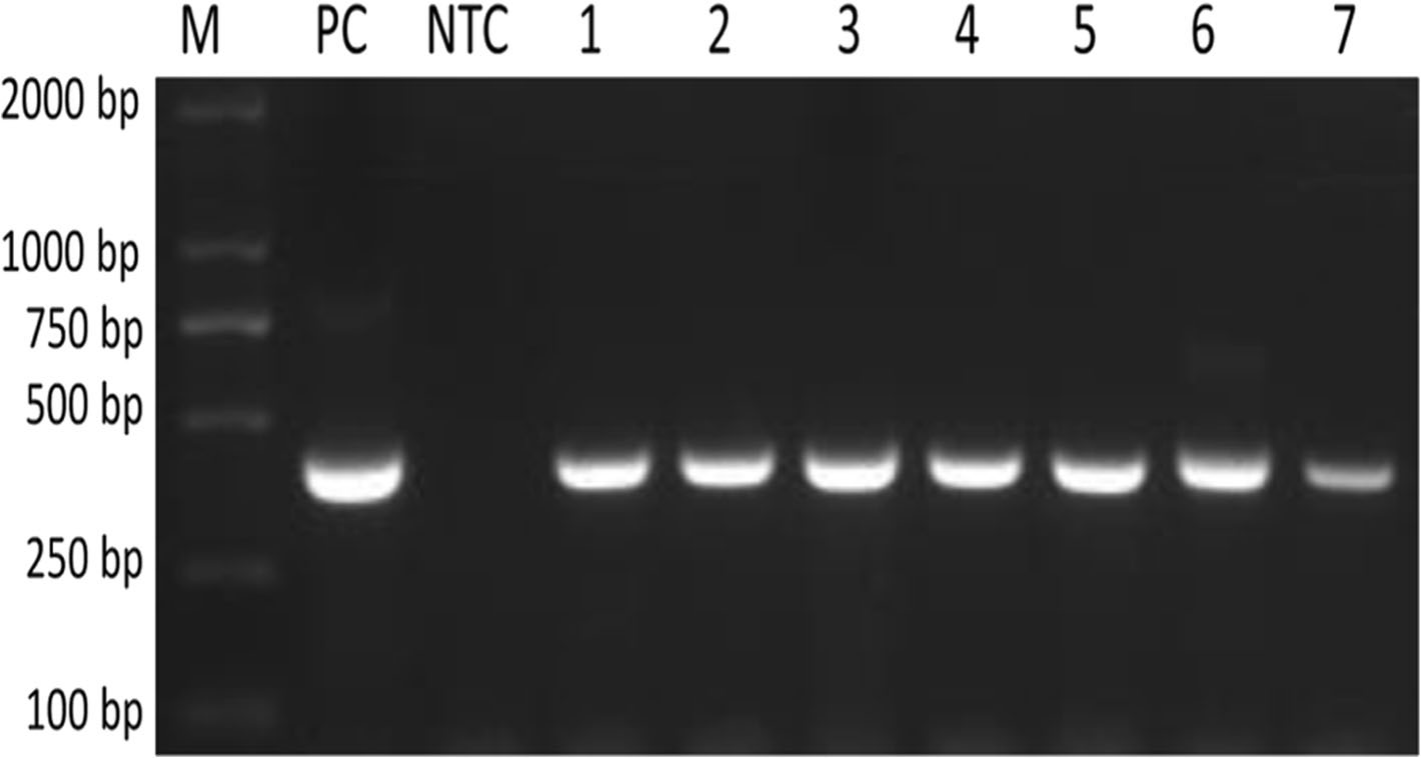
1.5% agarose gel electrophoresis image showing a 393 bp band for the amplification of the V4 region of the *18S* rRNA gene of piroplasm species. Lane M: DL 2000 DNA marker; Lane PC: positive control; Lane NTC: negative test control; Lanes 1–7: positive identified samples by semi-nested PCR

**Fig. 2:**
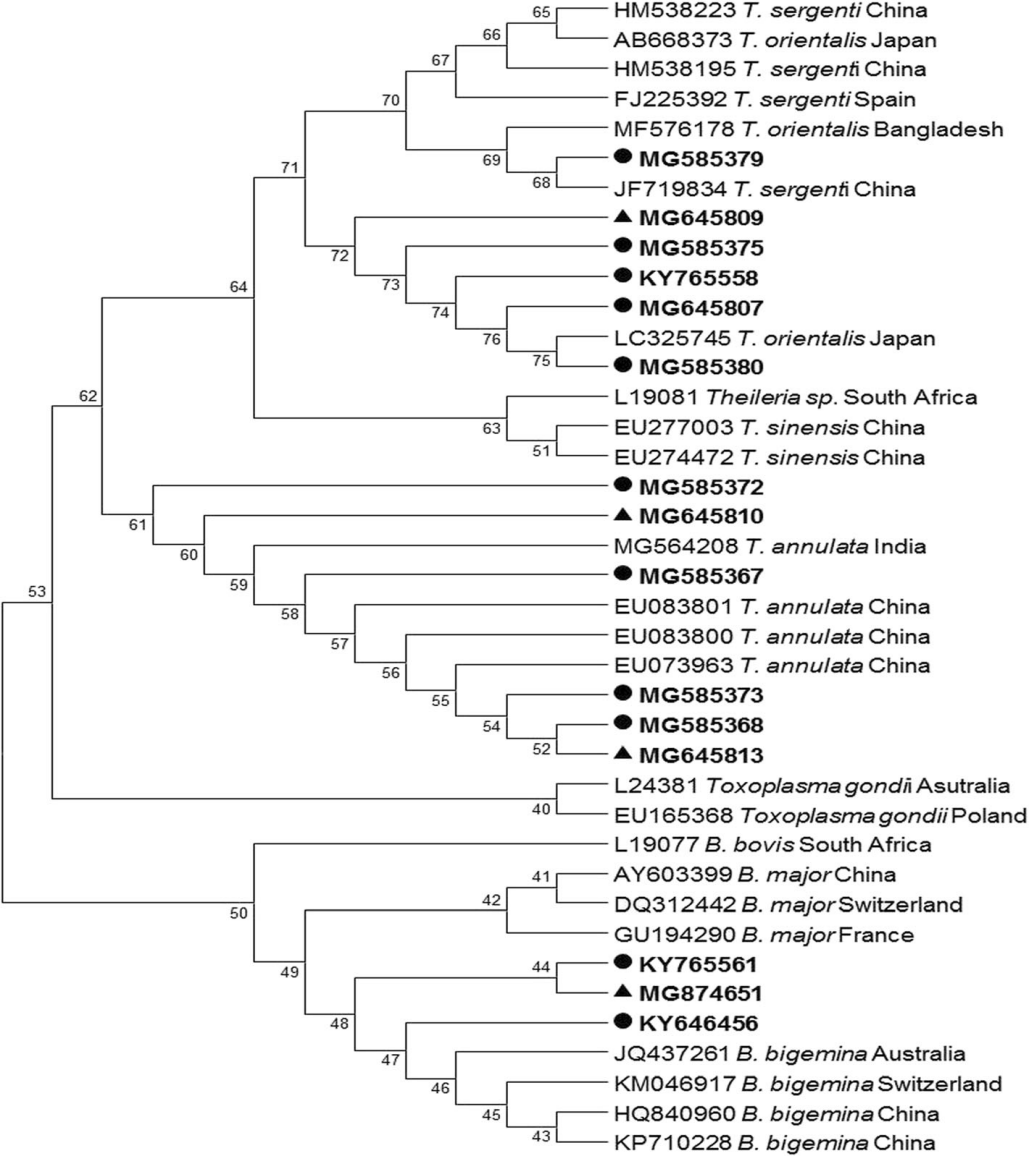
Phylogenetic tree for *Theileria* spp. and *Babesia* spp. based on the V4 region of the *18S* rRNA gene sequences. The parasites identified in the present study are marked in bold. Samples from Pakistan are indicated by circles; samples from China are indicated by triangles

Semi-nested PCR was used to detect *Theileria* and *Babesia* spp. by targeting the V4 region of the *18S* rRNA gene and amplifying a 393-bp fragment (Fig. 1). Of the 570 bovine blood samples, 144 (25.26%) samples were positive for piroplasms, showing an approximately 393-bp product band in the agarose gel. The PCR products of all the positive detected 144 samples were sequenced. The phylogenetic analysis based on the short amplified sequences of the *18S* rDNA V4 hypervariable region revealed three piroplasm species (*T. annulata*, *T. orientalis* and *B. bigemina*) in samples from the two study areas (Fig. 2). *Theileria annulata* (115/570; 20.17%) was the most prevalent piroplasm species. The highest prevalence of *T. annulata* was found in Chakwal (46.80%), followed by Faisalabad (10.75%), Inner Mongolia (10.0%) and Jhang (2.96%). The second most abundant species was *T. orientalis* (16/570; 2.80%). The highest prevalence of *T. orientalis* was found in Chakwal (6.38%), followed by Faisalabad (2.15%) and Inner Mongolia (1.67%). *Theileria orientalis* was not found in Jhang. *Babesia bigemina* (13/570; 2.28%) was the only species of *Babesia* found in any of the samples. The highest prevalence of *B. bigemina* was in Chakwal (5.31%), followed by Inner Mongolia (1.67%) and Faisalabad (1.07%); this species was not found in Jhang. The highest piroplasm overall prevalence was recorded in the samples from Chakwal (110/188; 58.51%), followed by Faisalabad (13/93; 13.97%), Inner Mongolia (16/120; 13.33%) and Jhang (5/169; 2.95%) (Table 2). The results of the long fragment amplification confirmed the presence of these species. The phylogenetic tree revealed the relationships between these species.

## Discussion

In Pakistan, small-holder cattle farming systems are common for both commercial and domestic purposes in resource-poor rural communities. In recent years, farmers have adopted commercial farming using modern techniques and imported breeds of cattle (*Bos taurus*) because of their higher milk yield potential. However, the imported animals are more prone to ticks and tick-borne diseases compared with the local breeds (*Bos indicus*), resulting in mortality rates as high as 80% in the former compared with < 20% in the latter animals. Among many tick-borne diseases, bovine theileriosis, predominantly caused by *T. annulata*, is considered among the most economically notorious diseases [5]. To date, most studies of bovine theileriosis have been conducted by using conventional microscopy, which, because of its low sensitivity and specificity, does not truly represent the actual distribution of the species involved. Recently, a study reported the prevalence of *T. orientalis* from imported and native bovine populations of Pakistan, describing 24.5% positive results in imported animals as compared with 6% positive results in native cattle [20]*.* In China, four *Babesia* species (*B. bovis*, *B. bigemina*, *B. major* and *B. ovata*) have been described as being responsible for cattle, buffalo and yak babesiosis [29, 30]. Recently, Liu et al. [30] detected a new bovine *Babesia*, similar to *B. venatorum* and Qin et al. [31] reported an epidemiological study of *B. orientalis* in China. Among different members of *Theileria*, *T. annulata*, *T. orientalis* and *T. sinensis*, have been reported to cause the disease in China [4]. Recently, *T. luwenshuni* was reported for the first time in yaks [32].

**Fig. 3:**
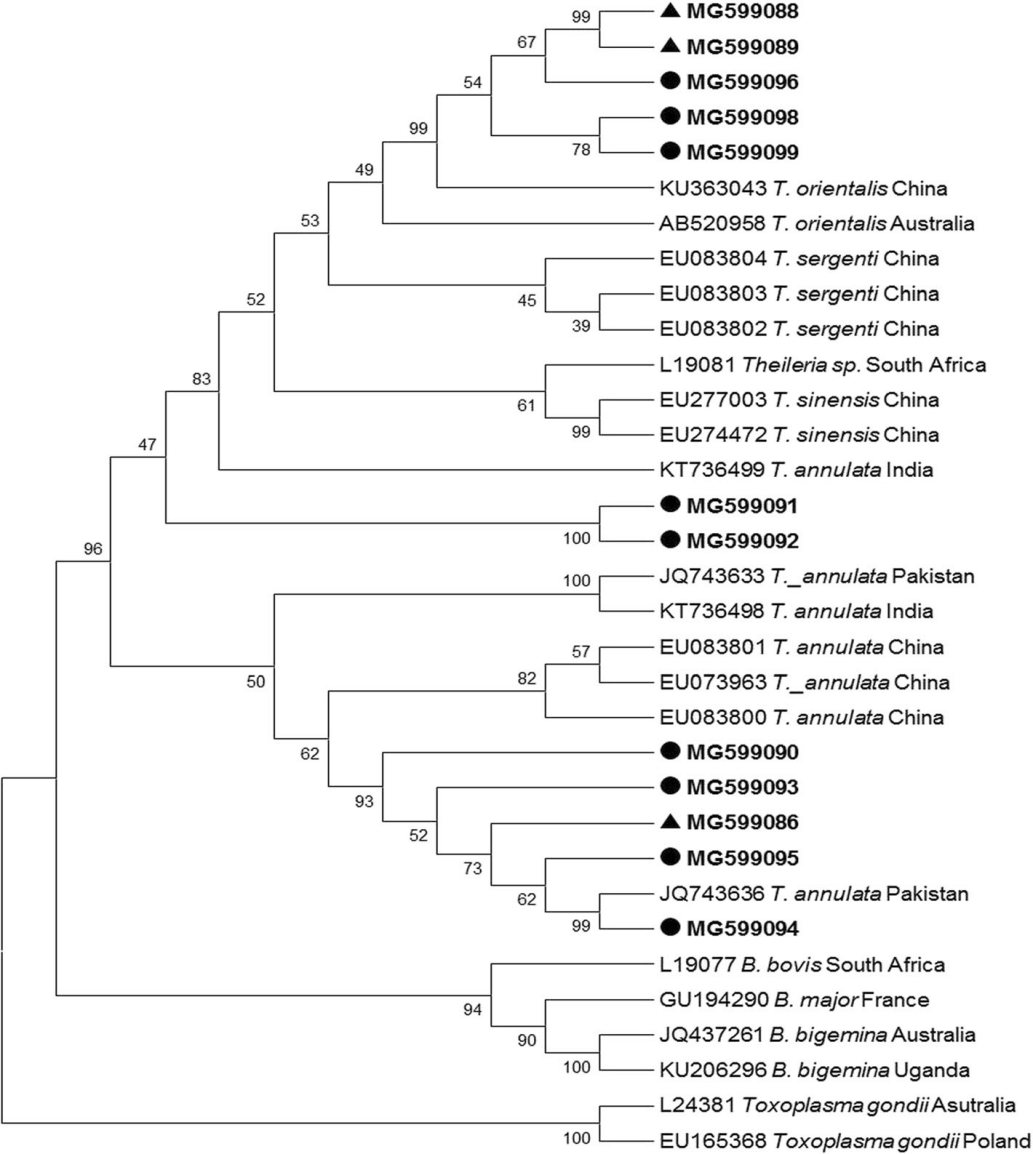
Phylogenetic tree for *Theileria* spp. constructed based on the *18S* rRNA gene sequences. The parasites identified in the present study are marked in bold. Samples from Pakistan are indicated by circles; samples from China are indicated by triangles

**Fig. 4:**
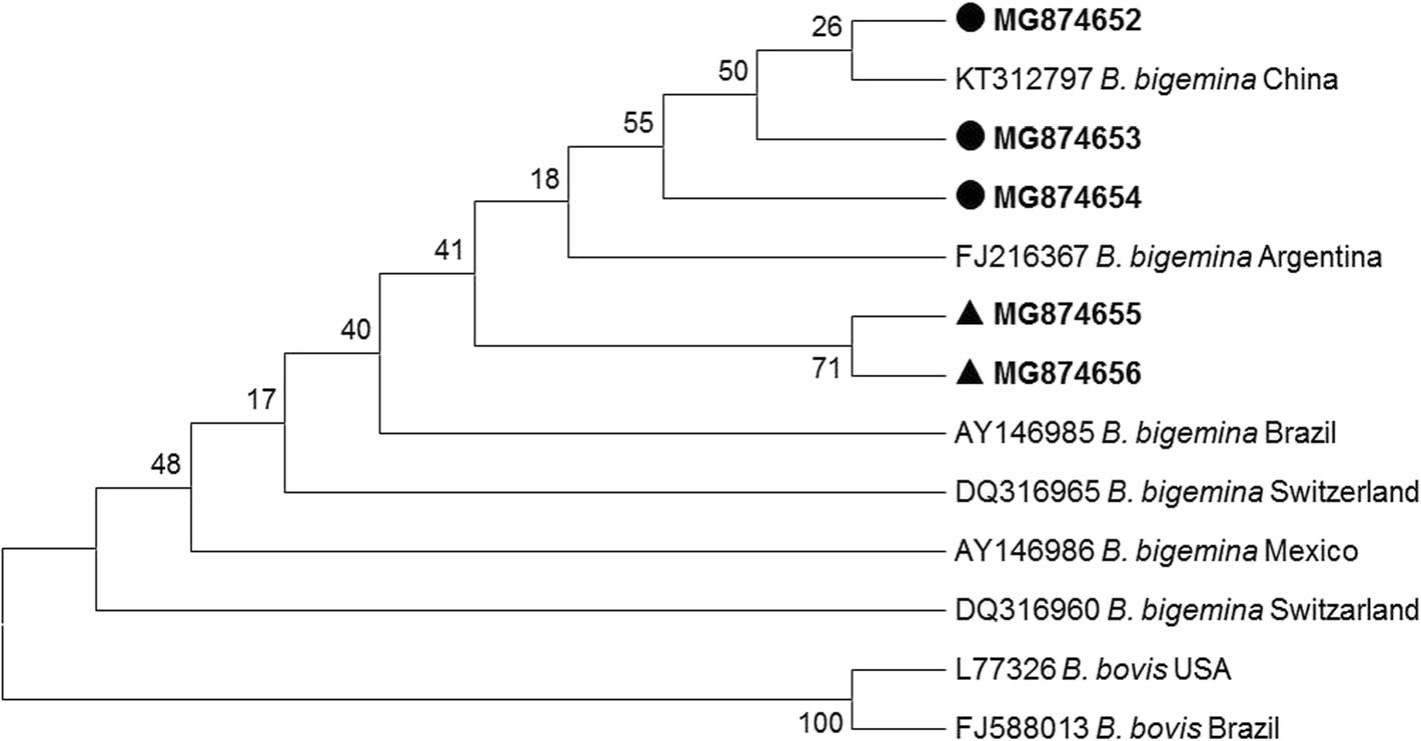
Phylogenetic tree for *Babesia bigemina* constructed based on the rhoptry associated protein (*RAP-1c*) gene sequences. The sequences identified in the present study are marked in bold. Samples from Pakistan are indicated by circles; samples from China are indicated by triangles

In the present study, phylogenetic analysis of *T. annulata* short sequences showed maximum similarity with sequences already reported from India and China, whereas, on the basis of long fragment amplified sequences, most of the *T. annulata* sequences were found to be closely related to sequences already reported from Pakistan [17] and Iran (GenBank: KF429799). A few short sequences of *T. orientalis* showed similarity with sequences already reported from China, whereas others showed a close relationship with sequences from Japan. Some of the long *T. orientalis* sequences reported from Pakistan were closely related to sequences already reported from China (GenBank: KU363043) and Pakistan (GenBank:JQ743636), whereas a few sequences were not closely related to sequences in GenBank and appear to be distinct (GenBank: MG599098 and MG599099) (Fig. 3). The sequence JQ743636 was obtained from gDNA of cattle imported from Australia to Pakistan. This shows that the disease in native animals might have come from infected imported animals. The *B. bigemina* short sequences were analyzed by using other reference sequences of the same gene [7]. These showed a relationship with reported sequences from Australia, China and Switzerland (Fig. 2). The long fragment sequences revealed a relationship with sequences reported from China and Argentina, with a few sequences occupying a distinct position in the phylogenetic tree (Fig. 4).

## Conclusions

The present study reports the simultaneous detection of multiple piroplasm species, and the genetic relationship between them, from selected areas of Pakistan and China. The results of this study could be helpful for providing a basis for vaccine production and control strategies for these economically important piroplasmosis.
